# Development of A Multi-Spectral Pyrometry Sensor for High-Speed Transient Surface-Temperature Measurements in Combustion-Relevant Harsh Environments

**DOI:** 10.3390/s23010105

**Published:** 2022-12-22

**Authors:** Sneha Neupane, Gurneesh Singh Jatana, Timothy P. Lutz, William P. Partridge

**Affiliations:** 1 Oak Ridge National Laboratory, Oak Ridge, TN 37830, USA; 2 Cummins Inc., Columbus, IN 47201, USA

**Keywords:** pyrometer, multi-spectral radiation thermometry (MRT), combustion, surface temperature

## Abstract

Accurate and high-speed transient surface-temperature measurements of combustion devices including internal combustion (IC) engines, gas turbines, etc., provide validation targets and boundary conditions for computational fluid dynamics models, and are broadly relevant to technology advancements such as performance improvement and emissions reduction. Development and demonstration of a multi-infrared-channel pyrometry-based optical instrument for high-speed surface-temperature measurement is described. The measurement principle is based on multi-spectral radiation thermometry (MRT) and uses surface thermal radiation at four discrete spectral regions and a corresponding emissivity model to obtain surface temperature via non-linear least squares (NLLS) optimization. Rules of thumb for specifying the spectral regions and considerations to avoid interference with common combustion products are developed; the impact of these along with linear and non-linear MRT analysis are assessed as a function of temperature and signal-to-noise ratio. A multi-start method to determine the MRT-solution global optimum is described and demonstrated. The resulting multi-channel transient pyrometry instrument is described along with practical considerations including optical-alignment drift, matching intra-channel transient response, and solution-confidence indicators. The instrument demonstrated excellent >97% accuracy and >99% 2-sigma precision over the 400–800 °C range, with ~20 µs (50 kHz, equivalent to 0.2 cad at 2000 RPM IC-engine operation) transient response in the bench validation.

## 1. Introduction

Accurate knowledge of in-cylinder surface temperatures plays an important role in heat-transfer modeling of IC engines and prediction of heat losses, which are relevant to overall engine efficiency, exhaust emissions, and component thermal stresses [1,2,3,4]. Specifically, surface temperature is a primary boundary condition for combustion computational fluid dynamics (CFD) or conjugate heat transfer (CHT) analysis, and directly impacts the resulting accuracy of cylinder heat-transfer losses and closed-cycle efficiency predictions; while sub-surface-temperature measurement is useful for referencing secondary sub-surface analysis predictions, given the unique dependence of CHT analysis on surface temperature, a direct measurement of this primary boundary condition is desired. Similarly, in gas turbine applications, accurate blade surface temperatures are critical to evaluating the effectiveness of cooling designs and for prolonging component thermal life [5,6,7]. Optical diagnostics are non-intrusive and allow fast (kHz rate) measurements of combustion transients such as temperature and species [8,9,10]. Such high-speed diagnostics can also be applied to implement real-time control strategies for reducing cycle-to-cycle variability in the combustion process [8,9,10,11,12,13]. 

An optical diagnostic based on multi-spectral infrared pyrometry for high-bandwidth measurements of transient surface temperatures within an operating combustor chamber has been developed and demonstrated, with the purpose of providing a direct measurement of the critical boundary condition for optimizing CHT analysis. The measurement principle is based on radiation pyrometry, which uses the spectral intensity of thermal radiation naturally emitted from the target surface to infer its temperature. Temperature can be determined using three different approaches of radiation thermometry: single wavelength, dual wavelength, and multi wavelength [14,15,16]. Multi-spectral or multi-wavelength radiation thermometry (MRT) uses emission-intensity measurements at four or more discrete wavelengths and an emissivity model to obtain an unknown surface temperature [14,15], and is preferred for its ability to enhance measurement accuracy and account for the complex spectral variations of both radiation intensity and emissivity [17,18]. 

Although successful thermocouple-based, surface-temperature-targeted measurements have been performed in IC engines [19,20,21], they are limited by the complexity of installation just below the target surface, lower time resolution due to slow temporal response, low accuracy due to radiative and conductive losses of thermocouple probes, and uncertainties associated with correlating the measurement to the actual surface temperature. Critically, thermocouples measure sub-surface temperatures and not the primary surface-temperature boundary conditions for CHT analysis. Gas-turbine-blade surface temperature is a strong function of the flow-field and thermocouple installation can disrupt the flow causing inaccurate temperature measurements [22]. Thermographic phosphors have been evaluated for zero- or two-dimensional surface-temperature measurements in engines and gas turbines [23,24,25,26,27]. Phosphor thermometry relies on the temperature-dependent changes in excitation-induced emission properties of ceramic materials doped with rare-earth or transition metals. The thermographic-phosphor transducing material must be coated on the device under test, and thus the measurement is of the phosphor overcoat rather than the native surface. An optical setup for both laser excitation and phosphorescence emission collection is required, which can be challenging in space-constrained IC engines. Hence, most phosphor thermography experiments have been performed in especially designed optically accessible research engines, where some parts of the engines were made of quartz or other optical material [24,25,26]. While such studies can undoubtedly provide valuable information, optical engines are inherently invasive in that they incorporate significant engine modification that change engine properties including surface temperature. Thus, the utility of phosphor thermography for providing accurate surface-temperature boundary conditions in native OEM metal engines can be limited by its use of a phosphor transducer and optical-access requirements.

Infrared-based pyrometry has been extensively researched and applied for measurements of flame temperature and soot distribution in combustion systems [13,16,28,29,30,31]. Monochromatic pyrometry involves the measurement of radiation intensity at a single wavelength (λ) and requires a knowledge of emissivity, ε_λ_, to solve for an unknown surface temperature. Two-color pyrometry uses a ratio of radiation intensities at two wavelengths (λ_1_ and λ_2_) to determine the unknown surface temperature and requires a knowledge of the surface emissivity ratio (ε_λ1_/ε_λ2_) at the two wavelengths. For soot pyrometry, spectral functions such as the A-method [15,16] or F-method [15,32] are used to model soot emissivity. When emissivities at the two measurement wavelengths (channels) are equal (i.e., gray body assumption, ε_λ1_ = ε_λ2_), the absolute surface temperature can be measured without knowledge of the absolute emissivity [15]. However, if the equivalent-emissivity (or gray body) assumption is not correct, two-color pyrometry can lead to errors [33]. Wavelength selection to make the gray body assumption for two-color pyrometry realistic (e.g., the two wavelength regions are sufficiently close) has been studied [34,35]. Multi-spectral thermometry involves measuring radiation intensity at four or more wavelengths and leverages least-squares techniques to simultaneously solve for temperature and surface emissivity [15]. Applications of two-color pyrometry to IC engines include surface-temperature measurements of diesel particulate filters [36] and diesel injector nozzles [37]. For gas turbine application, short (1 to 3 um) and longer (>10) wavelengths pyrometers [5,22] have been investigated. Dual-wavelength-ratio pyrometry has been proposed to account for errors due to reflected radiation at shorter wavelengths. Longer wavelength pyrometry has also been proposed due to high emittance of certain thermal barrier coatings at longer wavelengths [5]. Hence, wavelength selection for pyrometers is application specific and depends on the combustor-surface optical properties and interference from combustion gases.

This study focuses on the development of an infrared pyrometry-based optical instrument for high-speed (kHz rate) surface-temperature measurements. A key motivation is to enable direct surface-temperature measurements of combustion reactors with opaque metal/alloy surfaces such as IC engines for enhancing CHT and related engine-efficiency analysis. The instrument utilizes the MRT method to estimate surface temperature from spectrally resolved thermal radiation vs. the two-color methods used in previous IC-engine applications. The theory of the MRT method is summarized in Section 2. Linear and non-linear least squares techniques have been assessed for accurate determination of surface temperature from the MRT method, with the NLLS method selected (Section 3). Various design parameters that improve the accuracy of NLLS optimization were assessed and analyzed, e.g., the effect of the number, spread, and spacing of the spectral channels used in analysis (Section 4.1) and the positioning to mitigate interference with major combustion products (Section 4.2). Since the solution of the non-linear optimization can be sensitive to initial-guess values of temperature and emissivity, a multi-start method for selecting the MRT solution with minimum least squares error is proposed (Section 5). Hardware configuration of an optical probe and fiber-based multi-spectral pyrometer instrument is detailed along with the calibration method to account for instrument parameters and the MRT-analysis structure (Section 6). The steady state and transient precision, accuracy, and temporal resolution of the developed instrument is assessed via bench demonstration, along with additional practical considerations including detector settings, alignment state, and signal monitoring to assess MRT-solution confidence (Section 7).

## 2. Measurement Principle: Multi-Spectral Radiation Thermometry

Pyrometric non-contact temperature measurement techniques are based on Planck’s radiation heat transfer law (Equation (1)), which can be approximated as Wien’s law (Equation (2), applicable for *λ* << *c*_2_/*T*. For all configurations, the pyrometer requires calibration to determine the instrument factor of each measurement channel, which depends on geometrical factors, instrument optics, detector sensitivities, and other design parameters [16].

Planck’s Law:

(1) Lλ, Emλ, T=ελc1 λ5expc2λT−1 

$L_{\lambda ,\;Em}$: Emitted radiation intensity (W/m^2^-μm) at wavelength *λ* (μm) and temperature *T* (K)
$\varepsilon_{\lambda }$: Emissivity at wavelength *λ*
*c*_1_: 2π *h* c_o_^2^ = 3.742 E8 W-μm^4^/m^2^
*c*_2_: *h* c_o_/*k_B_* = 14,388 μm-K,

where *h* and *k_B_* are the Planck and Boltzmann constants, respectively, and c_o_ is the vacuum speed of light.

Wien’s Approximation:

(2) Lλ, Emλ, T=ελc1 λ5expc2λT  

Multi-spectral thermometry involves using measurements at four or more wavelengths and least-squares techniques to simultaneously solve for the unknown emissivity and surface temperature. For accurate determination of temperature using the MRT method, prior knowledge regarding the functional dependence of surface emissivity on wavelength is required; it typically includes simplifying assumptions that emissivity is continuous and single-valued over a specified wavelength region. Given these assumptions, emissivity can be approximated with any suitable spectral function, such as an exponential operating on a polynomial (exponential, Equation (3)) or a polynomial (Equation (4)).

(3) $$\varepsilon_{\lambda_{j}}=\;exp\;(a_{0}+a_{1}\lambda_{j}+a_{2}\lambda_{j}^{2}+\cdots +a_{m}\lambda_{j}^{m})$$

(4) $$\varepsilon_{\lambda_{j}}=(a_{0}+a_{1}\lambda_{j}+a_{2}\lambda_{j}^{2}+\cdots +a_{m}\lambda_{j}^{m})$$

In Equations (3) and (4), *m* is the order of the emissivity model and *j* represents the individual spectral regions used for the analysis. Linear and non-linear least squares techniques can then be applied to minimize the sum of the square of errors (SSE) between the calculated and measured radiation intensities to determine the unknown temperature and emissivity.

### 2.1. LLS MRT: Linear Least-Squares Method

When the fitting function for emissivity is chosen to be an exponential function of wavelength (Equation (3)), radiation intensities can be calculated from Wien’s approximation (Equation (2)) to obtain a set of linear equations that can be directly solved using the LLS or inverse technique to determine the best-fit temperature and emissivity. Rearranging Equation (2) and taking the natural logarithm of both sides, Wien’s approximation can be represented as:

(5) lnc1(Lλj.λj5)=c2λjT−lnελj,

where *j* represents the selected wavelengths and takes values from 1 to *n*. The left-hand side (LHS) of the Equation (5) is known, consisting of the measured *L_λ, Em_* and known *c*_1_ and *λ_j_* values; temperature and emissivity are the unknowns on the right-hand side (RHS). Representing the LHS as *N_λ__j_* and using the exponential emissivity function (Equation (3)) gives:

(6) $$N_{\lambda_{j}}=\frac{c_{2}}{T}\left(\lambda_{j}^{-1}\right)-\sum_{i=0}^{m}a_{i}.\lambda_{j}^{i},$$

where *i* = 0 to *m* (order of the exponential emissivity model). This system of equations can be represented in matrix form as:

(7) $$\left[\begin{array}{c} N_{\lambda_{1}}\\ N_{\lambda_{2}}\\ \vdots \\ N_{\lambda_{n}} \end{array}\right]=\left[\begin{array}{cccccc} 1 & \lambda_{1} & \lambda_{1}^{2} & \cdots & \lambda_{1}^{m} & \lambda_{1}^{-1}\\ 1 & \lambda_{2} & \lambda_{2}^{2} & \cdots & \lambda_{2}^{m} & \lambda_{2}^{-1}\\ \vdots & \vdots & \vdots & \cdots & \vdots & \vdots \\ 1 & \lambda_{n} & \lambda_{n}^{2} & \cdots & \lambda_{n}^{m} & \lambda_{n}^{-1} \end{array}\right]\left[\begin{array}{c} a_{0}\\ a_{1}\\ \vdots \\ a_{m+1} \end{array}\right]$$

where *n* represents the number of spectral regions used for the analysis. Equation (7) can be solved using linear algebra to calculate the best-fit least-squares solution of coefficients *a*_0_ to *a_m_*_+1_. Emissivity is then calculated from Equation (3) using *a*_0_… *a_m_* and Temperature from $T=\frac{c_{2}}{a_{m+1}}.$ The minimum number (*n_min_*) of wavelengths required for MRT analysis is *m* + 3, e.g., using a first-order exponential emissivity model (Equation (3) with *m* = 1) leads to three unknowns (*a*_0_, *a*_1_, and *T*) in Equation (7), and thus requires emitted-radiation-intensity measurements at four (*m* + 3) discrete wavelength regions for calculating the best-fit least-squares solution. Additional details on the LLS technique for determining the emissivity and temperature can be found in the literature [14,15].

### 2.2. NLLS MRT: Constrained Non-Linear Least-Squares Optimization Method

Emissivity can also be modeled as a polynomial (first- or higher-order) function of wavelength (Equation (4)). In this case, NLLS constrained optimization is applied to solve for temperature and emissivity. An *m*^th^-order polynomial (Equation (4)) is used to describe emissivity as a function of wavelength. Initial guess values for emissivity coefficients (*a*_0_–*a_m_*) and temperature (T) are used to calculate corresponding guess values of the emitted radiation intensity (*L_λ,ges_*) using Planck’s law (Equation (1)). Non-linear least squares optimization is then used to calculate the optimum solution for the emissivity coefficients and T by minimizing the SSE (Equation (8a)) between measured (*L_λ,meas_*) and guessed (*L_λ,ges_*) radiation intensities while constraining emissivity to between zero and unity (Equation (8b)). Additional details on the NLLS technique for determining emissivity and temperature can be found in the literature [14,15]. As with LLS MRT, NLLS MRT also requires radiation intensity measurements at a minimum of *m* + 3 wavelengths, where m is the order of the specified polynomial emissivity model (Equation (4)).

The objective function to minimize is:

(8a) $$\chi^{2}=\sum_{j=1}^{n}{\left\{L_{\lambda_{j},meas}-L_{\lambda_{j},ges}\right\}}^{2}\;$$

The linear emissivity model constraints are:

(8b) $$0\leq \varepsilon_{\lambda_{j}}\leq 1$$

## 3. Emissivity Model Selection

An emissivity model describing the functional dependence of surface emissivity on wavelength is required to accurately determine temperature using the MRT method. Emissivity models typically incorporate the simplifying assumptions that emissivity is continuous and single-valued over a defined wavelength range. With these assumptions, emissivity can be approximated using any suitable analytic function of wavelengths such as a polynomial (Equation (4)) or an exponential operating on a polynomial (Equation (3)). Wen et al. [17,38,39] investigated the effect of heating (or oxidation) on the surface emissivity of various aluminum and steel alloys, and the influence of the emissivity model order (m in Equations (3) and (4)) on the accuracy of predicted temperature using the MRT method. They concluded that for steel alloys, first-order (*m* = 1) models (polynomial or exponential) provide the most accurate results compensating for different alloys, surface oxidation conditions, and temperatures. For aluminum alloys, the authors found that the HHR (Hagen–Rubens relation, *ε* $=a_{0}\times {\left(\frac{T}{\lambda }\right)}^{\frac{1}{2}}$ [39]) emissivity model compensates well for oxidation variations and provides the best calculated-temperature accuracy. For both steel and aluminum surfaces, the authors found that higher-order (*m* = 2, 3…) emissivity models lead to decreased calculated-temperature accuracy. Another study [40] similarly concluded that using higher-order emissivity models can compromise calculation accuracy due to overfitting, even with very small deviations between the actual and modeled emissivity. Thus, the literature suggests that a first-order emissivity model provides the most accurate MRT temperature determination accounting for measurement noise, varying surface conditions due to heating and oxidation, and various aluminum and steel alloys.

**Figure 1:**
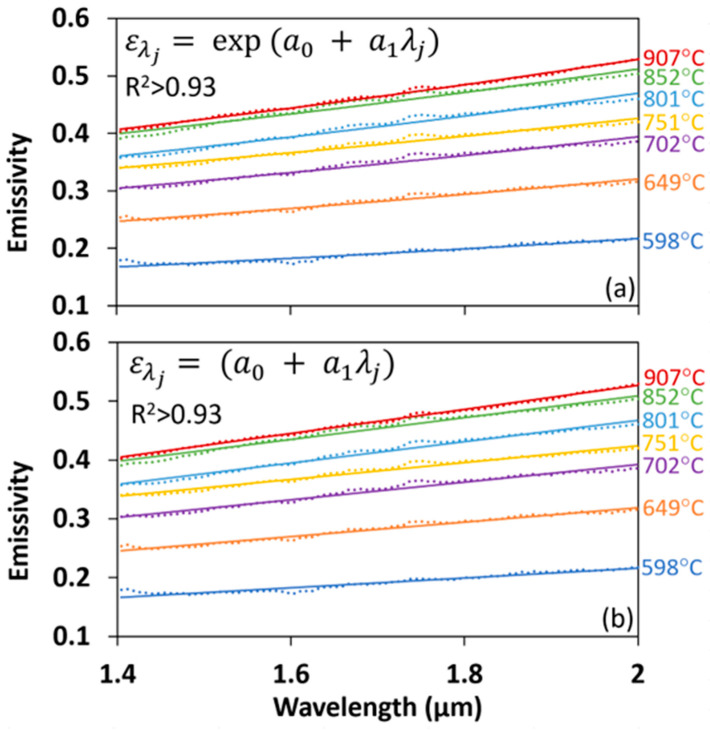
Measured emissivity (dotted curves) of a stainless-steel IC-engine valve and fits (solid curves) using a first-order (*m* = 1) (**a**) exponential and (**b**) polynomial emissivity model.

To confirm the suitability of a first-order emissivity model for the intended engine applications, the emissivity of a stainless-steel IC-engine valve surface at 500–850 °C was measured using a silica optical fiber (ThorLabs FT800EMT) and spectrometer (Ocean Optics: NIR256-2.5, wavelength range: 900–2550 nm). The spectrometer and the optical-fiber setup were first calibrated using a black-body source (Omega, BB-4A) and then used to measure the emitted radiation intensity of a valve sample placed in a furnace (see Section 6.2 for calibration details). The radiation intensity data were used to compute surface emissivity at different temperatures. Figure 1 shows the measured emissivity is well fit over the 1400–2000 nm range, for both the exponential (Equation (3)) and polynomial (Equation (4)) emissivity models using first order (*m* = 1), and that for this application, there is no need to use a higher-order emissivity model. Table S1 in the Supplemental Material summarizes the fitted emissivity coefficients and R^2^ values for the exponential and polynomial first-order emissivity models (Figure 1).

### 3.1. Linear vs. Non-Linear MRT

To guide instrument design, the applicability and impact of the exponential and polynomial emissivity models of Figure 1 on MRT analysis were assessed using synthetic emission data, which allowed for assessment over a wide range of signal-to-noise ratios (SNR). The results of this analysis do not represent performance of the developed instrument, which is discussed in Section 7, but it is performed as part of the instrument development to down-select between linear and non-linear MRT analysis. The synthetic emission data was generated via Planck’s law at seven standard temperatures (~600, 650, 700, 750, 800, 850, 900 °C) over the 1000–2000 nm wavelength region, with ~8 nm resolution; a first-order exponential or polynomial emissivity model (Table S1) was applied and random noise added to simulate instrument noise. Three different levels of random noise (2.5%, 5%, and 10%) were added to obtain datasets with different signal-to-noise ratios; the noise was generated using a sequency of random numbers (−1≤ # ≤+1) scaled using the target percentage of the average signal from the seven synthetic emission curves. Using such a fixed maximum noise amplitude across a wide range of signal levels (vs. scaled with signal level) was consistent with the nature of the spectrometer discussed in Section 3. The simulated data were averaged over a 40 nm wide spectral window (i.e., 5-point moving average) to obtain three additional noise levels for each emissivity model. Figure S1 in the Supplemental Material shows an example dataset using the polynomial emissivity model with 10% random noise and the corresponding 5-point moving-average noise. The result was 42 noise levels for each emissivity model, i.e., seven temperatures and six noise levels. The simulated data were used with the corresponding emissivity model to perform linear and non-linear least-squares (LLS and NLLS) MRT analysis using four (1400, 1600, 1800, 2000 nm) equally spaced spectral regions. For a given temperature and noise level, the average SNR was determined as the average value between the four analysis wavelengths. The result is 42 datasets over a range of temperatures and SNR values (~10–2400) for each LLS and NLLS MRT method, which are used here to down-select the emissivity model and in Section 4.1 to assess the number of spectral channels for MRT analysis.

Table 1 summarizes the error in MRT-calculated temperature using both NLLS and LLS emissivity models as a function of actual temperature and the average signal-to-noise ratio (SNR). The 42 individual SNR points for each model that are the basis of Table 1 are shown in Figure S2a, with Figure S2b graphically representing the average error results of Table 1. Both emissivity models produce excellent temperature measurements with <10% error when the spectral-channel-average SNR is ≥150, while the NLLS model has similarly low error across the investigated temperature range for 50 ≤ SNR ≤ 150. The NLLS model significantly outperforms the LLS model at lower SNR values, resulting in 83% and 54% lower error in the 50–150 and 0–50 SNR regions, respectively. Table 1 further breaks down the analysis among the seven analysis temperatures, highlights how the low-SNR and high-error points occur at lower temperatures where signals are lower, and further details the superior accuracy of the NLLS MRT analysis in the low-SNR region. The specific SNR of the developed instrument is discussed in Section 7. 

Based on the results of Table 1, non-linear MRT using a first-order (linear) polynomial emissivity model was selected for further analysis and development of the multi-spectral pyrometry instrument described in Section 6.1. Furthermore, we show in Section 7 (Bench Validation) that the linear-polynomial emissivity model is valid over the wider wavelength region (1200–3600 nm) selected for the four-bandpass pyrometry instrument described in Section 6.1. The following sections investigate the influence of spectral-channel parameters on MRT analysis (Section 4) and an initial-guess methodology for implementing NLLS MRT (Section 5). 

## 4. Wavelength Regions Selection

**Figure 2:**
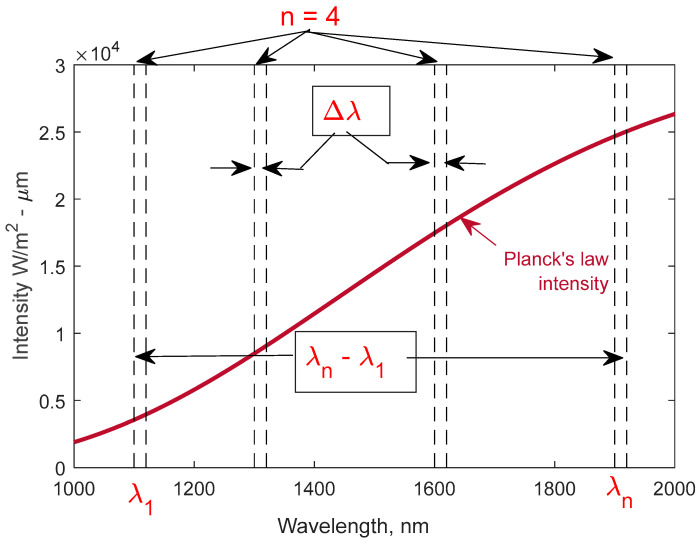
Wavelength parameters including number (*n*), spectral width or bandwidth (Δ*λ_j_*), and adjacent spacing (*λ_j_*–*λ_j_*_−1_), where *j*: 1 to *n* and total spectral range (*λ_n_*–*λ*_1_) of the different regions must be selected and influence MRT analysis performance.

The temperature calculated from MRT analysis is sensitive to the spectral-analysis parameters described in Figure 2, including the number of wavelength regions (*n*), spectral width of each region (Δ*λ_n_*), spacing of adjacent wavelength regions (*λ_j_*–*λ_j_*_−1_, where *j*: 1 to *n*), and total spectral range (*λ_n_*–*λ*_1_). Section 4.1 describes general rules of thumb for specifying these wavelength-selection parameters that we developed and found to improve MRT-analysis accuracy. Gas-phase absorption and emission between the emitting surface and collection optics can impact MRT analysis and wavelength-selection parameters, e.g., chemiluminescence flame and burned-gas emission, and absorption by combustion products. Section 4.2 describes wavelength-parameter specification to eliminate absorption and emission interference to MRT analysis in IC-engine applications. 

### 4.1. Wavelength Selection Rules of Thumb

The number of spectral regions required for MRT analysis is dictated by the number of unknowns, i.e., temperature and coefficients in the emissivity model. As described in Section 3, the minimum number of measurement wavelength regions or channels (*n_min_*) for calculating the best-fit emissivity coefficients and temperature (for either the LLS and NLLS emissivity model) is *m* + 3, where m is the order of the selected emissivity model. Hence, four spectra channels are required for a first-order emissivity model (*m* = 1). Several of the literature studies have indicated that increasing the number of spectral channels beyond *n_min_* does not significantly improve, and can actually degrade MRT accuracy [39,40], despite the commonly expected relationship of increasing samples reducing errors. To demonstrate the sufficiency of minimizing wavelength channels, NLLS MRT analysis was compared using four (1400, 1600, 1800, 2000 nm) and seven (1400, 1500, 1600, 1700, 1800, 1900, 2000 nm) equally spaced spectral regions, a linear (*m* = 1) emissivity model, and the simulated emission data and measurement-SNR calculation described in Section 3.1. The results of this analysis do not represent performance of the developed instrument, which is discussed in Section 7, but it is performed as part of the instrument development to down-select the number of spectral channels for MRT analysis. 

Table 2 summarizes the error in the NLLS MRT-calculated temperature using four and seven spectral channels (*n*) as a function of the average SNR based on the synthetic pyrometry data. The 42 individual SNR points for each n value, on which Table 2 is based, are shown in Figure S3a, while Figure S3b graphically represents the average error results of Table 2. Little practical difference is observed between using *n* = 4 and 7 when the spectral-channel-averaged SNR is ≥50, and both result in excellent temperature accuracy with <10% error above this SNR. While the *n* = 7 analysis provides ~25% lower error in the lowest 0–50 SNR region, both have relatively low accuracy in this range (~30–40% error). Figure S3a shows how even these high errors are due to 1–4 points (see points with Error >60% and SNR <12 in Figure S3a inset), and if not for those, even the low-SNR accuracy would be better. The temperature-specific details of Table 2 reveal little systematic benefit of increasing the spectral channels beyond *n_min_*. Moreover, implementations beyond *n_min_* correspondingly expand the instrument complexity, cost (detectors, data-acquisition channels, etc.), size, data management, computational time, potentially transient response, and/or SNR (e.g., if the channels are sequentially cycled through, which might limit channel dwell times), etc., which creates a motivation to minimize the spectral channels. Considering this and the results of Table 2, NLLS MRT using four spectral channels (*n_min_*) was selected for development of the multi-spectral pyrometry instrument described in Section 6.1.

**Figure 3:**
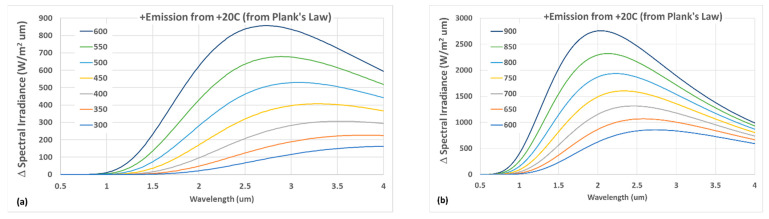
Additional spectral emission from a +20 °C surface temperature change in (**a**) relatively low-T (300–600 °C) and (**b**) high-T (600–900 °C) ranges. Calculated via Planck’s law.

The location and range of the selected wavelength regions influences MRT analysis sensitivity. Figure 3 shows additional surface emission associated with a +20 °C temperature increase at selected temperatures in the low-T (300–600 °C, Figure 3a) and high-T (600–900 °C, Figure 3b) ranges. The curves were calculated using Planck’s law and represent spectral sensitivity of the emission to a +20 °C temperature increase, wherein sensitivity is directly proportional to the curve height. Since the sensitivity follows Wien’s displacement law, the peak sensitivity shifts to lower wavelengths at higher temperatures. Figure 3a demonstrates how a low-T range is most sensitive in the ~2–4 μm range, with peak sensitivity shifting from ~4 to 2.75 μm with increasing temperature. In contrast, sensitivity in the high-T range (Figure 3b) degrades through the 3–4 μm region and is highest in the ~1.5–3 μm range, i.e., at shorter wavelengths than for a low-T diagnostic. Typically, diagnostics are designed for a specific application range and with corresponding tradeoffs, with Figure 3 demonstrating a challenge of making a high-sensitivity instrument over a very broad temperature range. Other factors and tradeoffs such as component (lenses, optical fibers, filters, detectors, etc.) transmission, sensitivity, noise, cost, etc., also influence wavelength selection and diagnostic design, with some of these aspects addressed in Section 4.2. For this study, the target of measuring a minimum surface temperature of 300–400 °C guided the diagnostic design to include wavelength measurements in the 2.5–4 μm range to enhance low-T sensitivity. More generally, measurements at higher and lower wavelengths enhance lower- and higher-temperature sensitivity, respectively.

In addition to the spectral range (*λ_n_*–*λ*_1_), the distribution of the measurement wavelengths within that range also impacts MRT accuracy. Non-linear MRT analysis was performed on the synthetic emission data with 10% noise (described in Section 3) at four analysis temperatures (610, 714, 819, 911 °C), with the *λ_n_* values spread over narrow (1.5, 1.6, 1.7, 1.8 μm) and broader (1.0, 1.4, 1.8, 2.0 μm) spectral ranges. Table 3 shows that the average temperature error is six times lower with the broader spectral-channel distribution, i.e., 0.20 vs. 1.24 for the broader and narrow *λ_n_*–*λ*_1_ ranges, respectively. Comparing this to Figure 3b suggests that the sensitivity gradient across the *λ_n_*–*λ*_1_ range is important in addition to absolute sensitivity among the *λ_n_* channels; specifically, the net absolute sensitivity among the *λ_n_* channels appears greater in the narrow (1.5–1.8 μm) distribution, but the sensitivity gradient among the *λ_n_* channels is greater for the broader (1.0–2.0 μm) distribution. While we have not comprehensively studied the tradeoffs between sensitivity levels and gradients, Table 3 indicates that broadly distributing the *λ_n_* regions over a range capturing both high sensitivity and gradients improves MRT accuracy.

### 4.2. Wavelength Selection to Avoid Measurement Interference from Combustion Gases

Multiple gas species involved in the combustion process have emissions and absorption bands in the visible and infrared regimes, and surface-pyrometry measurements must avoid these potentially interfering emission and/or absorption processes. For instance, in-cylinder IC-engine gas-phase emission has been used to study ignition and combustion processes, air-to-fuel ratio (AFR) transients and distributions, knock, and combustion control [41,42,43,44,45,46,47]. Transient gas emission measurements using spectrally broad (~200–650 nm) integration can have a bimodal temporal shape, with the initial mode arising from flame-front emission and the second mode due to emission from the burned-gas region behind the flame front [43,45]. The flame-front emission sweeps through the optical sampling field first; the burned-gas emission signal follows and can be larger due to its greater spatial extent and more extensive overlap with the optical field of view, while in other cases, reactions controlling the burned-gas emission can be quenched by relatively cool cylinder surfaces [43,45]. Flame-front emission is spectrally distinct from OH* (~306 nm), CH* (~390 and 432 nm), and the C2* Swan bands (~432, 470, 516, and 560 nm) in the near UV and visible [43,44,45,46,47,48]. Emission from the burned-gas region is spectrally broad and indistinct (~380–475 nm), consistent with that from a CO + O_2_ combustion (H_2_ + CO_2_ ↔ CO + H_2_O; 2CO_2_ ↔ 2CO + O_2_), and identified as arising from CO_2_ [45,46,47]; this emission follows the combustion pressure transient via its cascading impact on gas temperature and kinetic rates of the reactions controlling the emission. Of these UV-visible emission sources, CO_2_ is relevant to the IR region used for the pyrometry measurements reported here. As CO_2_ is also a major combustion product, it is considered separately as a potentially interfering absorbing species.

Gas-phase absorption can also impact the surface-pyrometry measurements, with the spectral regions used carefully selected to avoid this interference source. Major combustion products relevant to the pyrometry measurements have been measured [41,42,49,50]; these include major combustion products such as H_2_O (~1.1–1.2, 1.3–1.5, 1.7–2.0, 2.4–3 μm) and CO_2_ (~2.7–2.8, 3.9–4.6 μm), formaldehyde or H_2_O_2_ intermediates (3.2–3.8 μm), aldehydes (3.6–3.7 μm), and fuels (3.3–3.5 μm) (spectral bands are noted parenthetically). By selecting spectral-integration regions outside the spectral bands of these major combustion products, interference from related absorption as well as emission from both the flame front and burned-gas region are avoided in the pyrometry measurements. 

**Figure 4:**
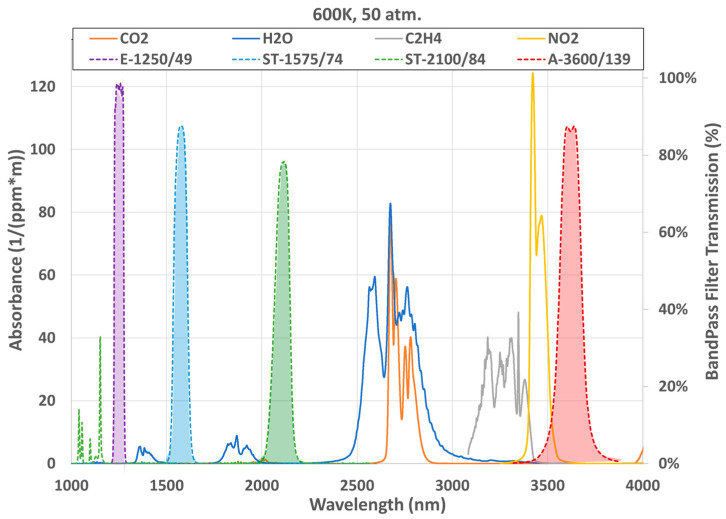
Absorbance of major combustion products and spectral regions defined by available bandpass filters to avoid related interference in the pyrometry instrument. The C_2_H_4_ region is generally representative of hydrocarbon fuels.

Four bandpass filters (λ_center_ = 1250, 1575, 2100, 3600 nm) were selected for the pyrometry instrument using MRT analysis, and to avoid interference from absorption bands of the major combustion product species, as well as emissions from the flame and burnt-gas species; the specific filters are discussed in Section 6.1 and detailed in Table S2. Figure 4 shows the transmission profile of the four spectral filters among major combustion product absorption features at 50 atmospheres and 600 K calculated using the HITRAN database [51]. While the absorption spectra clearly limit candidate filter spectral locations, the selected wavelength regions practically eliminate interference from flame and burnt-gas emission and absorption; this setup may be vulnerable to aldehyde and formaldehyde interference, which could be monitored in the engine exhaust via FTIR to select suitable interference-free, engine-operating conditions. As discussed in Section 4.1 in relation to Figure 3, the three low-wavelength regions are most suitable for high-temperature measurements, while the 3600 nm channel enhances low-temperature sensitivity. The zirconium fluoride (ZrF_4_) optical fiber used to connect the sapphire-fiber optical probe to the instrument (detailed in Section 6) imposes a long-wavelength limit of ~4.5 μm on range for selecting a candidate spectral filter. Using a 4670-nm bandpass filter (Edmund, 150-nm FWHM, 84-074) on the red side of the fundamental CO_2_ absorption feature, in combination with switching to an indium fluoride (InF_3_, ~5.5 μm cutoff) patch fiber, could further enhance low-temperature sensitivity and avoid formaldehyde and aldehyde interference.

## 5. Multi-Start Method to Find Global Optimum

The NLLS optimization solution is sensitive to the initial-guess values of temperature and emissivity coefficients, in addition to the spectral-channel parameters discussed in Section 4. Since most non-linear programming methods have been developed with the aim of finding the local minimum, using different initial-guess values can lead the non-linear objective function to converge to different local minima. There may exist several local minima, and the corresponding function values may differ substantially. In this study, we use the multi-start approach [52] which involves conducting local minimization from a set of starting points distributed over the feasible temperature domain, and then choosing the solution corresponding to a minimum function value. The multi-start approach is a well-known stochastic approach and has been researched widely in the literature [52,53]. Studies have also proposed methods to select the set of feasible initial-guess values, their distribution, etc. [53,54]. Several other variants have been reported in the literature, such as multi-start with clustering [52,55], domain elimination [56], zooming [56], and repulsion [57]. The basic multi-start method can be inefficient as it requires multiple executions of the local search, and particular minima may be located several times. The development or selection of the most accurate and efficient global optimization technique is outside the scope of this study. Hence, we use the basic multi-start method with initial temperature guesses varying from 500–1400 °C in 50 °C intervals, and choose the solution corresponding to the minimum function value. The initial guess for emissivity coefficients (*a*_0_ and *a*_1_ of linear emissivity model: $\varepsilon_{\lambda_{j}}=a_{0}+a_{1}.\lambda_{j}$) was kept at 0.1 and 0.02, respectively, for all optimizations. 

Table 4 and Figure S4 show implementation of the multi-start approach used in this study to determine an optimum temperature solution using data from a 911.3 °C (1184 K) surface; these steady state measurements used the spectrometer described in Section 3 and the engine-valve sample and setup described in Section 7. Nineteen initial-temperature guesses (500–1400 °C at 50 °C intervals, as shown in Table 4, and corresponding to guesses 1:19 on the *x*-axis of Figure S4) were used to solve the optimization problem. For each initial-temperature guess, the pyrometry instrument calibration factors (Section 6.2) and selected emissivity model (Section 3) were used to predict a corresponding signal at each of the four wavelength bands; the resulting sum of the squared error (SSE) between these values and the corresponding signals from the measured spectral channels was calculated, and the initial-temperature guess with the lowest SSE was selected as the optimum solution. Table 4 shows the MRT solution along with numerical SSE and error values resulting from each initial guess; the rows corresponding to initial guesses resulting in low SSE and temperature error are highlighted in green and those with high values are highlighted in red. The multi-start optimization converges to solutions around 910 °C and 980 °C with low and high SSE, respectively. As expected, the absolute error in the calculated temperature is lower for solutions with lower SSE (or objective function value); SSE ranges from ~0.001 to 0.04 for absolute temperature errors of <0.1% vs. ~1.8 when the absolute temperature error is >7%. Interestingly, low-SSE and low-error (accurate) solutions are not necessarily clustered around initial guesses near the accurate temperature solution, or adjacent to low-SSE initial guesses (e.g., Guess # 13–17). While guesses 1–5, 14, and 16 (500–700, 1150, 1250 °C) all converge to low-SSE and high-accuracy solutions, the 1150 °C initial-guess solution has the lowest SSE (0.001), and this minimum-SSE, 910.8 °C, 99.9% accurate case is chosen as the MRT solution. In Section 7 (Bench Validation), we further validate this multi-start method for our target temperature range of interest and demonstrate an accuracy of >97%.

## 6. Instrument Setup and Calibration

Using the design guidelines from the preceding sections, an optical probe and multi-spectral pyrometry instrument are developed (Section 6.1), calibration is described and implemented (Section 6.2), and the structure of the MRT-analysis script is described (Section 6.3). 

### 6.1. Optical Probe and Instrument Hardware

A forward-viewing optical probe was developed to gather surface emission for spectral measurements via the instrument and MRT analysis to determine surface temperature. The probe was based on a 425 μm diameter sapphire optical fiber (Photran LLC, Poway, CA, USA) housed in a Hastelloy C tube (1/16 in OD, 0.020 in ID, VICI: Valco Instruments Co. Inc., Houston, TX, USA), and mounted in an SMA fitting.

**Figure 5:**
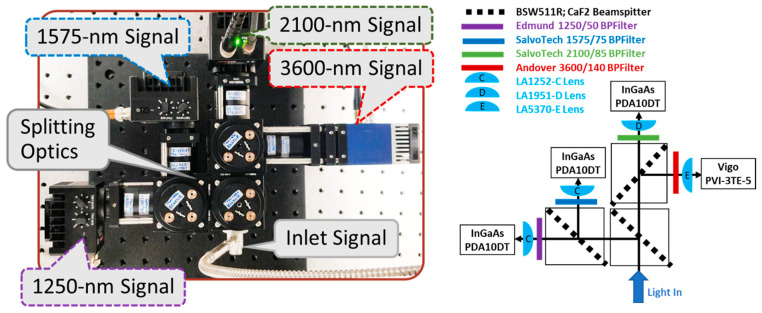
Pyrometry instrument setup picture and schematic detailing part numbers.

Figure 5 shows the pyrometry instrument, which measures surface emission via four spectral channels centered at 1250, 1575, 2100, and 3600 nm, and provides data for MRT analysis to determine surface temperature. Light from the optical probe is transported to the instrument via a zirconium fluoride (ZrF_4_) optical fiber (ThorLabs MZ61L1, 600 mm diameter, 0.20 NA, Newton, NJ, USA) and collimated using a parabolic mirror (ThorLabs, RC02SMA-P01). The instrument is based on cage-system hardware and uses three 50/50 CaF_2_ beamsplitters (ThorLabs, BSW511R) to create the four equal-intensity spectral channels. Each of the four beams passes through selected bandpass filters and focusing lenses (Table S2) prior to their respective detectors. Thermoelectrically cooled indium–gallium–arsenide (InGaAs) detectors (ThorLabs, PDA10DT) were used for the 1250, 1575, and 2100 nm channels, and a thermoelectrically cooled photovoltaic detector (Boston Electronics, Vigo PVI-3TE-5, Brookline, MA, USA) with improved longer-wavelength response was used for the 3600 nm channel. The signals (Volts) from the four detectors were collected using a high-speed data acquisition system (NI DAQ: PXIe-6366 and BNC-2110) and LabView software. Analysis involved converting the voltage signals to radiation intensity units (W/m^2^-μm) using pre-determined calibration factors (Section 6.2) and implementing non-linear MRT fitting (Section 6.3) to solve for surface temperature. 

### 6.2. Instrument Calibration

Calibration accounts for the spectrally varying instrument response (attributable to the optical probe and fiber, various optical components, and detectors) and conversion of detector signals (V) to emitted radiation intensity (W/m^2^-μm, see Equation (1)). A black-body source (Omega, BB-4A) was used with the instrument and forward-viewing probe to determine calibration functions for each channel. Calibration measurements were made at nine temperatures (~400 to 800 °C at 50 °C intervals). The black-body temperature vs. measured signal at each spectral channel is well fit using a power law (Figure S5); the fit functions (R^2^ > 0.98) are shown in Table S3, with their form structured to convert measured signals (e.g., S_1250nm_ (V)) at each channel to equivalent or apparent black-body temperature (e.g., T_a,1250nm_ (K)). Apparent temperature is defined as the temperature of a black body which emits the same radiation intensity as the non-black body (real surface) at temperature T [16]. The initial step of calibrating measured signals from a real surface of unknown temperature involves applying the calibration functions to determine the equivalent apparent temperature for each spectral channel (T_a,λ_). Planck’s law (Equation (1)) for a black body (ε_λ_ = 1) is then used with each T_a,λ_ and λ_c_ pair to calculate radiation intensities (*L_λ,Em_*(λ,T) (W/m^2^-μm)) at the apparent temperature for each wavelength channel. The radiant intensities are then used with an appropriate emissivity model to calculate the surface temperature via Equations (1) or (2). In practice, these measured radiation intensities are used in NLLS MRT analysis to determine the surface temperature of the emitting surface.

### 6.3. MRT Analysis

**Figure 6:**
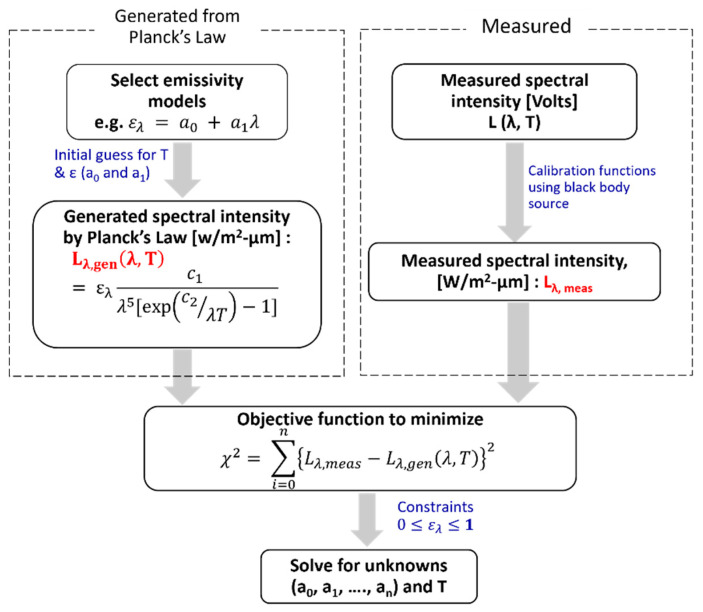
Solution optimization schematic for NLLS MRT.

Multi-spectral radiation thermometry analysis theory, for both LLS and NLLS techniques, has been described in Section 2, and the suitability of a first-order emissivity model and wavelengths selected have also been justified in Section 3 and Section 4. In this section, we summarize the MRT parameters and analysis method used for temperature calculations from measured raw detector signals. The first-order linear emissivity model was chosen and NLLS optimization was used. Since it was determined that the minimum number of required wavelengths is sufficient for an accurate temperature calculation, four wavelengths (1250, 1575, 2100, 3600 nm, Table S2) were selected to avoid interference from combustion-gas emission and absorption. Figure 6 shows a schematic of the MRT solution process. The pyrometry instrument described in Section 6.1 was used to measure signal intensity at the four spectral channels (in Volts); calibration factors were then used to convert the measured intensities to apparent temperature, and then to equivalent black-body radiation intensities (W/m^2^-μm), as described in Section 6.2. Multi-start non-linear constrained optimization was subsequently used to minimize the SSE between the Measured (*L_λ,meas_*) and Generated (*L_λ,gen_*) radiation intensities at the four wavelengths, i.e., minimize SSE between the upper-right and left dashed boxes in Figure 6. Equation (8a) is the objective function to minimize and the linear inequality constraints for emissivity are given in Equation (8b) (Section 2.1). The optimization was conducted using the multi-start approach (Section 5), with initial guess values of temperature ranging from 500–900 °C at 50 °C intervals, and the temperature solution corresponding to the minimum SSE was selected. 

## 7. Bench Validation of the Instrument and the MRT Analysis

**Figure 7:**
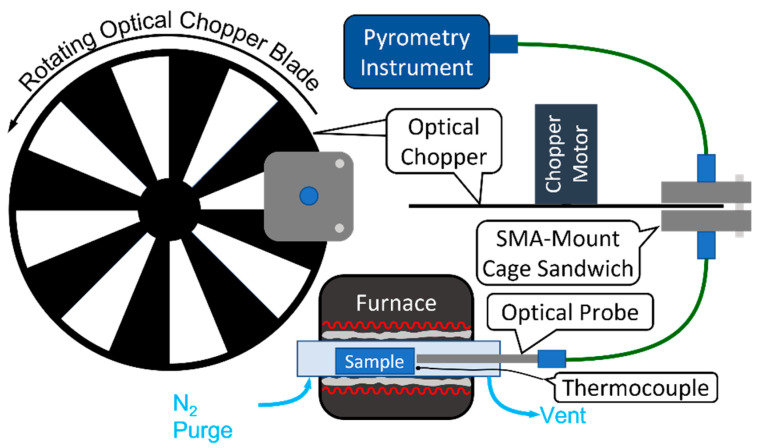
Schematic of laboratory bench setup for measuring surface temperatures of an LNF exhaust valve, and optical-chopper assembly for creating synthetic surface-emission transients to assess transient response of the pyrometry instrument. The furnace assembly houses the valve sample in an N_2_-purged quartz tube; insulation (gray) shields the sample and optical probe from the furnace heating elements (red); a thermocouple measures environment temperature near where the optical probe captures surface emission. The chopper assembly is inserted in the optical path between the probe and pyrometry instrument.

The pyrometry instrument and MRT analysis method were validated using laboratory bench measurements of the surface temperatures of an exhaust valve from a 2.0 L GM Ecotec LNF engine under steady state (SS) and transient surface-emission conditions, at a range of temperatures; this engine setup has been detailed in the literature [58] and is the intended platform for future engine demonstrations. As shown in Figure 7, the stainless steel LNF valve was placed in a quartz tube housed in a temperature-controlled furnace (Lindberg Blue, 55035) with inert (N_2_) purging and insulation (McMaster-Carr 93315K51) to reduce spatial gradients and shield the valve sample and optical probe from the furnace heating elements; the sapphire-fiber optical probe tip was positioned 5 mm from the valve sample, with the ZrF_4_ patch fiber connecting the probe to the pyrometry instrument, which was used to measure the surface radiation intensities at different temperatures. The true surface temperature of the LNF valve was measured using a thermocouple inserted into the furnace and positioned near the point of measured surface emission. Surface radiation was measured once the sample-environment temperature had reached SS. i.e., when the furnace set-point temperature was steady and approximately equal to the measured thermocouple temperature. 

The MRT-analysis solutions at seven SS temperatures from 400 to 800 °C are shown in Table 5, along with the true temperatures (400, 448, 501, 553, 601, 697, 802 °C). The measurements were made at 2 MHz (100 k samples in 50 ms) and averaged to 20 kHz (1 k samples in 50 ms) prior to MRT analysis; the MRT results were equivalent for both the 20 kHz averaged and native 2 MHz data. The 20 kHz averaged-data MRT analysis provides 100 µs resolution, which is equivalent to 1.2 crank-angle degrees (cad) at 2000 RPM and sufficient to resolve intra-valve-event transients. The detectors for the 1250 nm, 1575 nm, and 2100 nm channels were set to 50, 50, and 60 dB gain, respectively; the detector for the 3600 nm channel did not have selectable gain. The four-bandpass pyrometry instrument provided excellent SS measurements with >97% accuracy and >99% 2-sigma precision over the 400–800 °C range, as shown in Table 5. The channel-specific (Table S4) and average SNR values monotonically increase with temperature; the values vary from channel to channel, with the 3600 nm channel systematically having the lowest value and the average values varying from ~20–3500. Despite the utility of the synthetic data in guiding instrument design in Section 3.1 and Section 4.1, the bandpass pyrometry instrument has very low noise and can thus provide excellent measurements, even with a channel-averaged SNR~60; 2-sigma noise in the raw (mV) signal channels was ~0.2–0.6% at 500 °C and ~0.06–0.2% at 600 °C. The excellent results of Table 5 are for a well-calibrated instrument, though results will degrade as the instrument becomes misaligned relative to the calibration condition; the results of Table 5 are from experiments and calibration performed within the same week. Over time and with instrument movement, the individual instrument channels can suffer independent degrees of misalignment. For instance, similar data taken a month after calibration had similar noise but ~ 60%, 50%, 40% lower, and 30% higher signal levels for the 1250, 1575, 2100, and 3600 nm channels, respectively, while using the same calibration as in Table 5 resulted in degraded ~97.2% and 96.7% accuracy at ~500 and 600 °C, respectively. While this accuracy remains high, the MRT results will degrade with further misalignment between the calibration and application conditions. To mitigate these errors, the alignment should be optimized in application conditions to match reference channel-specific signal levels from a standard (e.g., black body) source recorded during calibration.

To assess the pyrometry instrument transient response independent of practical material-heating limitations, the constant-temperature surface emission was modulated using an optical chopper. Figure 7 shows a schematic of the setup used to create synthetic surface-emission transients. An optical chopper (SRS SR540) was positioned in the gap of an SMA-mount cage sandwich (ThorLabs CP33T, SM1SMA, ER1), across which the surface emission was pitched; the gap was aligned with and slightly wider than the chopper blade to minimize emission-signal losses and chopper-blade contact with the SMA-cage sandwich. This assembly was positioned between the optical probe and instrument. The chopper blade was rotated at fixed speeds to create synthetic surface-emission transients. While this approach provides a way to practically assess instrument transient response, the transition to and from full signal blocking (Blank) can result in anomalous MRT solutions which would not exist in actual surface-emission transients, e.g., the opaque edge moving through the emission beam can have a differing impact on the various channels due to the channel-specific optical alignment, while the impact of the different instrument channels drops below minimum-signal thresholds. These secondary details are demonstrated in the results below. We used a similar method to quantify instrument response during the early development of a fast absorption-based instrument for measuring transient EGR variations in an engine intake manifold [59,60]. 

**Figure 8:**
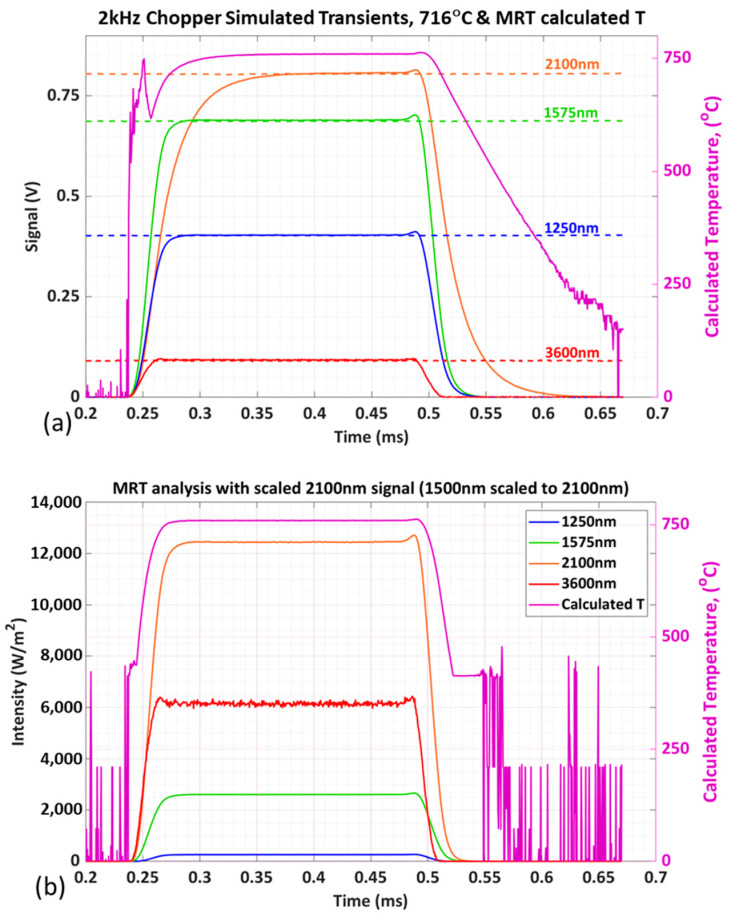
(**a**) Synthetic 2 kHz surface-emission transient from the LNF-valve sample at 716 °C using the chopper assembly shown in Figure 7. Individual signal transient from the four spectral channels and related transient MRT analysis results. Dashed lines are the signals at a steady state (without chopper). (**b**) Individual signal transient from the four spectral channels, with the slow 2100 nm channel replaced by a scaled version of the fast 1500 nm channel signal transient, and related transient MRT analysis results.

Figure 8a shows the response of the pyrometry instrument’s four spectral channels to a 2 kHz surface-emission transient from the LNF-valve sample at 716 °C and the resulting transient MRT analysis. Measurements were made at 2 MHz and with detector gains as noted with the SS analysis; a 16-bit data acquisition (DAQ) system (National Instruments PXI2-6366; 0.2 mV resolution on ±5 V scale) was used, with analysis performed using the MATLAB optimization toolbox. The 2 kHz chopped square-wave transient is cleanly resolved in Figure 8a, with near half-period (0.25 ms) high and low sections corresponding to the open and opaque portions of the optical-chopper blade. All channels reach steady high-level signals for significant portions of the half-period with >97% accuracy and >99% precision equivalent to the SS benchmarking, and the MRT solution drops in the transitions from and to full-signal blocking. The cost or tradeoff of a higher gain for the 2100 nm detector is apparent in its slower response (Figure 8a) and demonstrates the importance of balancing gain and transient response; matching this gain to that of the lower-wavelength channels would provide equivalent high transient response and signal levels well resolved by the DAQ. Furthermore, the slower transient response of the 2100 nm channel significantly degrades the transient response of the temperature solution, and notably causes the rising and falling transients to differ significantly. The onset transient is relatively fast because all channels have a significant signal level, although the slow 2100 nm channel causes some onset-transient anomalies. In contrast, the MRT solution in the transient tail is much slower because the fast-channel signals go to zero while the slow-channel signal remains and approaches zero at later times; this trend of the MRT solution to converge to progressively lower temperatures as the channel signals progressively drop below a threshold value, as is apparent in Figure 8a, has been consistently observed in Blank conditions and is discussed more in relation to Figure 8b. Nevertheless, even in this non-optimized configuration, Figure 8a shows that the instrument resolves 37–115 μs onset transients (74–230 points at 2 MHz), which is equivalent to 0.4–1.4 cad at 2000 RPM and is suitable for resolving transients within individual intake and exhaust valve events [9].

To investigate optimizing and matching transient response between the different spectral channels, the slow 2100 nm signal was replaced by a scaled version of the fast 1500 nm signal, as shown in Figure 8b; in practice, this would be achieved by using the same 50 dB detector gain for the three lower-wavelength channels. The resulting MRT temperature solution transients are faster, better behaved, and with matching rising and falling edges. The rising and falling transients occur over ~20 µs, which is equivalent to 0.2 cad at 2000 RPM. This demonstrates the importance of balancing the transient response of the different spectral channels, and with such balancing, the 4-channel pyrometry instrument is capable of resolving fast surface-temperature transients relevant to engine-combustion research.

Anomalous MRT solutions near Blank conditions are apparent in Figure 8 and demonstrate the need to establish channel-specific signal thresholds for assessing confidence in the MRT solution. Clearly, the noisy 0 °C-average solutions at full-Blank conditions are anomalous, but Figure 8b shows an apparently steady but clearly anomalous ~400 °C solution in the transition to Blank. The 1250 and 3600 nm channels go to zero at approximately 0.51 s in Figure 8b, and so even this ~600 °C solution in the tailing transient would be questioned. The solution temporarily stabilizes at ~40 °C in the transient tail at ~0.52 s, near when the 1575 nm signal drops to almost zero, and then transitions to full-Blank solutions at approximately 0.546 s when all channels are at Blank conditions. In practice, the channel-specific thresholds should be established and referenced in the analysis script to assess confidence in the MRT solution; in general, all channel signal levels should be above these thresholds to fully accept the MRT solution. 

## 8. Conclusions

The methodology for developing a multi-spectral pyrometry instrument for transient temperature measurement of IC-engine in-cylinder surfaces is described and implemented, with the accuracy, precision, and transient response of the resulting instrument demonstrated. Linear and non-linear least-squares MRT analysis theory is described along with the corresponding emissivity model fits to IC-engine valve samples per the intended application; the performances of the different methodologies are assessed over a range of temperatures and SNR levels, with a non-linear MRT scheme selected for data analysis. Rules of thumb regarding the number, bandwidth, spacing, and spread of the spectral channels are developed, with a four-channel instrument plan selected. Additional spectral-channel considerations to avoid interference with common combustion products are also discussed and incorporated. A multi-start method to determine the MRT-solution global optimum is described and demonstrated. Using the calibration and MRT analysis approach and the results of the design analysis, an instrument and optical probe are developed. Bench demonstration is used to assess the instrument performance and highlight the importance of maintaining instrument alignment relative to the calibration state, matching the transient response of the various spectral channels, and monitoring their signal levels vs. thresholds for assessing MRT-solution confidence. The four-channel MRT-pyrometry instrument demonstrated excellent >97% accuracy and >99% 2-sigma precision over the 400–800 °C range, with ~20 µs (50 kHz, 0.2 cad at 2000 RPM) transient response in the bench validation.

## Figures and Tables

**Table 1:** Average error in MRT-calculated temperature at different SNR regions (W/m^2^-µm) using NLLS and LLS methods and the synthetic surface-emission data. The columns break down the results as a function of the seven analysis temperatures and on an average basis across all analysis temperatures. Cells with ‘-’ indicate that the data of Figure S2a,b did not provide points in the corresponding range.

Average SNR	MRT Fitting Method	Average % Error In MRT-Calculated Temperature							
		600 °C	650 °C	700 °C	750 °C	800 °C	850 °C	900 °C	Average
0–50	LLS	180	46.5	75.4	48.3	-	-	-	87.6
	NLLS	66.6	14.1	6.9	75.1	-	-	-	40.7
50–150	LLS	15.0	258	24.0	14.4	9.7	34.5	1.9	51.1
	NLLS	0.56	11.8	25.0	10.6	2.6	5.7	4.7	8.7
150–250	LLS	-	-	4.6	7.4	3.1	-	2.4	4.4
	NLLS	-	-	0.9	9.2	4.6	-	3.0	4.4
250–500	LLS	-	6.0	-	-	0.1	6.4	5.4	4.5
	NLLS	-	-	-	-	4.9	5.4	5.1	5.1
>500	LLS	-	-	-	7.5	-	3.7	3.9	5.0
	NLLS	-	1.2	-	5.8	-	5.7	5.0	4.4

**Table 2:** Error in MRT-calculated temperature at range of SNR values (W/m^2^-µm) using four and seven spectral channels (*n* = 4 and 7) and the synthetic surface-emission data.

Average SNR	*n*	Average % Error In MRT-Calculated Temperature							
		600 °C	650 °C	700 °C	750 °C	800 °C	850 °C	900 °C	Average
0–50	4	66.6	14.1	6.9	75.1	-	-	-	40.7
	7	10.6	14.5	21.9	72.5	31.3	-	-	30.2
50–150	4	0.6	11.8	25.0	10.6	2.6	5.7	4.7	8.7
	7	0.8	3.8	30.7	7.9	4.8	4.4	9.6	8.9
150–250	4	-	-	-	9.2	4.6	-	3.0	5.6
	7	-	-	-	4.3	4.6	-	4.8	4.6
250–500	4	-	-	0.9	-	4.9	5.4	5.1	4.1
	7	-	2.2	5.0	5.8	3.8	5.7	5.4	4.6
>500	4	-	1.2	-	5.8	-	5.7	5.0	4.4
	7	-	-	-	-	-	5.8	5.0	5.4

**Table 3:** Error in calculated temperature (T) from NLLS MRT analysis using (a) narrow and (b) broader distribution of the four measurement wavelengths.

Analysis T (°C):		610	714	819	911	Avg.
Error % in Calculated T	(Narrow *λ* region) Selected *λ*: 1.5, 1.6, 1.7, 1.8 um	1.37	1.29	1.19	1.12	1.24
	(Broader *λ* region) Selected *λ*: 1.0, 1.4, 1.8, 2.0 um	0.34	0.21	0.14	0.09	0.20

**Table 4:** MRT-calculated temperature, SSE values, and corresponding error in calculated temperature using 19 different initial guesses for temperature, based on pyrometry measurements of a 911.3 °C (1184 K) surface, as described in Section 7. The colors are to highlight Guess # leading to solutions with low (green) or high (red) SSE.

Guess #	Initial Guess T (C)	Initial Guess T (K)	MRT Calculated T (C)	SSE	Error in MRT Calculated T (%)
1	500	773.2	910.5	0.039	−0.09
2	550	823.2	910.5	0.040	−0.09
3	600	873.2	910.5	0.031	−0.09
4	650	923.2	910.5	0.036	−0.09
5	700	973.2	910.5	0.036	−0.09
6	750	1023.2	979.1	1.803	7.44
7	800	1073.2	979.3	1.779	7.46
8	850	1123.2	979.3	1.781	7.46
9	900	1173.2	979.3	1.781	7.46
10	950	1223.2	979.2	1.783	7.46
11	1000	1273.2	979.4	1.776	7.47
12	1050	1323.2	979.1	1.799	7.44
13	1100	1373.2	979.3	1.776	7.47
14	1150	1423.2	910.8	0.001	−0.06
15	1200	1473.2	979.4	1.776	7.47
16	1250	1523.2	910.5	0.036	−0.09
17	1300	1573.2	979.4	1.776	7.47
18	1350	1623.2	979.2	1.786	7.45
19	1400	1673.2	979.4	1.776	7.47

**Table 5:** Steady state MRT results (MRT-T) from the pyrometry instrument at seven temperatures (True-T) based on laboratory bench measurements. Average SNR (W/m^2^-µm) values are shown (Table S4 includes channel-specific SNR values). The results are based on an analysis of 20 kHz averaged data, which provides 100 µs resolution or 1.2 cad at 2000 RPM. The bold is intended to highlight that particularly relevant line.

True-T (°C)	400	448	501	553	601	698	802
MRT-T (°C)	394	449	506	565	616	715	819
% Error	1.5	0.2	1.1	2.2	1.6	2.4	2.3
**Accuracy (%)**	**98.5**	**99.8**	**98.9**	**97.8**	**98.4**	**97.6**	**97.7**
2-Sigma Precision (%)	99.72	99.84	99.92	99.96	99.97	99.99	99.96
SNR(Average)	63	137	265	488	815	1858	2492

## Data Availability

The data on which this work was based is described and quantified in the text, tables and figures of this publication and its related supplemental material.

## Supplementary Materials

The following supporting information can be downloaded at: https://www.mdpi.com/article/10.3390/s23010105/s1, Figure S1: Simulated emission data at seven standard temperatures using Plank’s Law, a 1st-order (*m* = 1) polynomial emissivity model, with 10% random noise, and a 5-point moving average; Figure S2: (a) Scatterplot of LLS and NLLS MRT analysis error at 41 SNR levels corresponding to the analysis in Section 3.1. The horizontal dashed line is at 10% temperature error, and vertical dashed lines are shown at SNR of 50 and 150. Inset shows all points, and how both NLLS and LLS MRT have <10% error at very high (~2300) SNR, and how LLS has some very high (~500–630%) at low (~60) SNR. In general, both emissivity models result in <10% temperature error for SNR > 150. (b) Average error in MRT-calculated temperature from LLS and NLLS MRT analysis for different SNR regions (W/m^2^-µm) using the synthetic surface-emission data. Figure S3: (a) Scatterplot of NLLS MRT analysis error using 4 & 7 spectral channels at 41 SNR levels corresponding to the analysis in Section 4.1. The horizontal dashed line is at 10% temperature error, and vertical dashed lines are shown at SNR of 120. Inset shows all points, and some high (~70–150%) at low (~60) SNR. In general, <10% temperature error is achieved for both 4 & 7 spectral channels for SNR>120. (b) Error in NLLS MRT-calculated temperature using the synthetic data set described in Section 3.1 and four and seven spectral channels (i.e., n = 4 & 7) over a range of SNR values (W/m^2^-µm). Figure S4: SSE values and corresponding error in calculated temperature using 19 different initial guesses for T. X-axis represents the count for initial guess value of temperature (1 to 19 for T range of 500–1400 °C at 50 °C interval). Figure S5: Calibration curves showing signal (V) from each spectral channel at a given black-body temperature (K). Table S1: Fitted emissivity coefficients using exponential and linear models. Table S2. Center wavelength (*λc*), full-width at half maximum (FWHM) and source information for the instrument bandpass filters, and focal length (*f*) and source for the focusing lenses of the instrument. Table S3. Power-law calibration fits for converting measured signal (e.g., S_1250nm_) at each spectral channel to apparent blackbody temperature (e.g., T_a,1250nm_). Table S4. Channel-specific and average SNR (W/m^2^-µm) values are show. Based on analysis of 20-kHz averaged data, which provides 100-µs resolution or 1.2 cad at 2000 RPM.

## Notice

This manuscript has been authored by UT-Battelle, LLC under Contract No. DE-AC05-00OR22725 with the U.S. Department of Energy. The publisher, by accepting the article for publication, acknowledges that the United States Government retains a non-exclusive, paid-up, irrevocable, worldwide license to publish or reproduce the published form of this manuscript, or allow others to do so, for United States Government purposes. The Department of Energy will provide public access to these results of federally sponsored research in accordance with the DOE Public Access Plan.

## References

[1] Kundu P., Scarcelli R., Som S., Ickes A., Wang Y., Kiedaisch J., Rajkumar M. (2016). Modeling Heat Loss through Pistons and Effect of Thermal Boundary Coatings in Diesel Engine Simulations Using a Conjugate Heat Transfer Model.

[2] Margot X., Quintero P., Gomez-Soriano J., Escalona J. (2021). Implementation of 1D–3D integrated model for thermal prediction in internal combustion engines. Appl. Therm. Eng..

[3] Broatch A., Olmeda P., Margot X., Escalona J. (2019). New approach to study the heat transfer in internal combustion engines by 3D modelling. Int. J. Therm. Sci..

[4] Iqbal O., Arora K., Sanka M. (2014). Thermal map of an IC engine via conjugate heat transfer: Validation and test data correlation. SAE Int. J. Engines.

[5] Manara J., Zipf M., Stark T., Arduini M., Ebert H.-P., Tutschke A., Hallam A., Hanspal J., Langley M., Hodge D. (2017). Technology, Long wavelength infrared radiation thermometry for non-contact temperature measurements in gas turbines. Infrared Phys. Technol..

[6] Arulprakasajothi M., Rupesh P.L. (2022). Surface temperature measurement of gas turbine combustor using temperature-indicating paint. Int. J. Ambient. Energy.

[7] Kerr C., Ivey P.J. (2004). Optical pyrometry for gas turbine aeroengines. Sens. Rev..

[8] Jatana G., Geckler S., Koeberlein D., Partridge W. (2017). Design and development of a probe-based multiplexed multi-species absorption spectroscopy sensor for characterizing transient gas-parameter distributions in the intake systems of IC engines. Sens. Actuators B Chem..

[9] Jatana G., Kocher L., Moon S.-M., Popuri S., Augustin K., Tao F., Wu Y., Booth R., Geckler S., Koeberlein D. (2018). Mapping of exhaust gas recirculation and combustion-residual backflow in the intake ports of a heavy-duty diesel engine using a multiplexed multi-species absorption spectroscopy sensor. Int. J. Engine Res..

[10] Jatana G.S., Perfetto A.K., Geckler S.C., Partridge W.P. (2019). Absorption spectroscopy based high-speed oxygen concentration measurements at elevated gas temperatures. Sens. Actuators B Chem..

[11] Soid S., Zainal Z. (2011). Spray and combustion characterization for internal combustion engines using optical measuring techniques—A review. Energy.

[12] Sementa P., Vaglieco B.M., Catapano F. (2012). Thermodynamic and optical characterizations of a high performance GDI engine operating in homogeneous and stratified charge mixture conditions fueled with gasoline and bio-ethanol. Fuel.

[13] Drake M., Haworth D. (2007). Advanced gasoline engine development using optical diagnostics and numerical modeling. Proc. Combust. Inst..

[14] Mansoor A., Allemand C., Eagar T. (1991). Noncontact temperature measurement. II. Least squares based techniques. Rev. Sci. Instrum..

[15] Araújo A. (2017). Multi-spectral pyrometry—A review. Meas. Sci. Technol..

[16] Zhao H., Ladommatos N. (1998). Optical diagnostics for soot and temperature measurement in diesel engines. Prog. Energy Combust. Sci..

[17] Wen C.-D., Mudawar I. (2002). Experimental investigation of emissivity of aluminum alloys and temperature determination using multispectral radiation thermometry (MRT) algorithms. J. Mater. Eng. Perform..

[18] Luan Y., Mei D., Shi S. (2021). Light-field multi-spectral radiation thermometry. Opt. Lett..

[19] Marr M.A., Wallace J.S., Chandra S., Pershin L., Mostaghimi J. (2010). A fast response thermocouple for internal combustion engine surface temperature measurements. Exp. Therm. Fluid Sci..

[20] Assanis D.N., Badillo E. (1989). Evaluation of alternative thermocouple designs for transient heat transfer measurements in metal and ceramic engines. SAE Trans..

[21] Chang J., Filipi Z., Assanis D., Kuo T., Najt P., Rask R. (2005). Characterizing the thermal sensitivity of a gasoline homogeneous charge compression ignition engine with measurements of instantaneous wall temperature and heat flux. Int. J. Engine Res..

[22] Kerr C., Ivey P. (2002). An overview of the measurement errors associated with gas turbine aeroengine pyrometer systems. Meas. Sci. Technol..

[23] Husberg T., Gjirja S., Denbratt I., Omrane A., Aldén M., Engström J. (2005). Piston Temperature Measurement by Use of Thermographic Phosphors and Thermocouples in a Heavy-Duty Diesel Engine Run under Partly Premixed Conditions.

[24] Kashdan J.T., Bruneaux G. (2011). Laser-Induced Phosphorescence Measurements of Combustion Chamber Surface Temperature on a Single-Cylinder Diesel Engine.

[25] Fuhrmann N., Litterscheid C., Ding C.-P., Brübach J., Albert B., Dreizler A. (2014). Cylinder head temperature determination using high-speed phosphor thermometry in a fired internal combustion engine. Appl. Phys. B.

[26] Fuhrmann N., Schneider M., Ding C., Brübach J., Dreizler A. (2013). Two-dimensional surface temperature diagnostics in a full-metal engine using thermographic phosphors. Meas. Sci. Technol..

[27] Heyes A., Seefeldt S., Feist J. (2006). Two-colour phosphor thermometry for surface temperature measurement. Opt. Laser Technol..

[28] Stojkovic B.D., Fansler T.D., Drake M.C., Sick V. (2005). High-speed imaging of OH* and soot temperature and concentration in a stratified-charge direct-injection gasoline engine. Proc. Combust. Inst..

[29] Matsui Y., Kamimoto T., Matsuoka S. (1980). A study on the application of the two–color method to the measurement of flame temperature and soot concentration in diesel engines. SAE Trans..

[30] Matsui Y., Kamimoto T., Matsuoka S. (1979). A study on the time and space resolved measurement of flame temperature and soot concentration in a DI diesel engine by the two-color method. SAE Trans..

[31] Musculus M.P., Singh S., Reitz R.D. (2008). Gradient effects on two-color soot optical pyrometry in a heavy-duty DI diesel engine. Combust. Flame.

[32] Howell J.R., Siegel R. (1971). Thermal radiation heat transfer. Volume 3-Radiation transfer with absorbing, emitting, and scattering media. https://ntrs.nasa.gov/api/citations/19710021465/downloads/19710021465.pdf.

[33] Müller B., Renz U. (2001). Development of a fast fiber-optic two-color pyrometer for the temperature measurement of surfaces with varying emissivities. Rev. Sci. Instrum..

[34] Marx D.T., Policandriotes T., Zhang S., Scott J., Dinwiddie R.B., Wang H. (2001). Measurement of interfacial temperatures during testing of a subscale aircraft brake. J. Phys. D Appl. Phys..

[35] Hijazi A., Sachidanandan S., Singh R., Madhavan V. (2011). A calibrated dual-wavelength infrared thermometry approach with non-greybody compensation for machining temperature measurements. Meas. Sci. Technol..

[36] Kong J., Shih A.J. (2002). Infrared Thermometry for Diesel Exhaust Aftertreatment Temperature Measurement.

[37] Ciatti S.A., Blobaum E.L., Foster D.E. (2002). Determination of Diesel Injector Nozzle Characteristics Using Two-Color Optical Pyrometry.

[38] Wen C.-D. (2011). Study of steel emissivity characteristics and application of multispectral radiation thermometry (MRT). J. Mater. Eng. Perform..

[39] Weng K.-H., Wen C.-D. (2011). Effect of oxidation on aluminum alloys temperature prediction using multispectral radiation thermometry. Int. J. Heat Mass Transf..

[40] Orlande H.R., Fudym O., Maillet D., Cotta R.M. (2011). Thermal Measurements and Inverse Techniques.

[41] Splitter D., Reitz R., Hanson R. (2010). High efficiency, low emissions RCCI combustion by use of a fuel additive. SAE Int. J. Fuels Lubr..

[42] Rein K.D., Sanders S.T., Lowry S.R., Jiang E.Y., Workman J.J. (2008). In-cylinder Fourier-transform infrared spectroscopy. Meas. Sci. Technol..

[43] Geiser F., Wytrykus F., Spicher U. (1998). Combustion Control with the Optical Fibre Fitted Production Spark Plug.

[44] Longdill S., Raine R., Blanchard G., Wright W.J.S.T. (2002). Investigation into air-fuel ratio measurement of a high performance two-stroke engine by an optical method. J. Engines.

[45] Chou T., Patterson D.J. (1995). In-cylinder measurement of mixture maldistribution in a L-head engine. Combust. Flame.

[46] Withrow L., Rassweiler G.M. (1931). Spectroscopic studies of engine combustion. Ind. Eng. Chem..

[47] Withrow L., Rassweiler G.M. (1938). Studying engine combustion by physical methods a review. J. Appl. Phys..

[48] Bleekrode R., Nieuwpoort W.C. (1965). Absorption and Emission Measurements of C2 and CH Electronic Bands in Low-Pressure Oxyacetylene Flames. J. Chem. Phys..

[49] Frank J.H., Barlow R.S., Lundquist C. (2000). Radiation and nitric oxide formation in turbulent non-premixed jet flames. Proc. Combust. Inst..

[50] Brookes S., Moss J.J.C. (1999). Flame, Measurements of soot production and thermal radiation from confined turbulent jet diffusion flames of methane. Combust. Flame.

[51] Gordon I.E., Rothman L.S., Hill C., Kochanov R.V., Tan Y., Bernath P.F., Birk M., Boudon V., Campargue A., Chance K.V. (2017). The HITRAN2016 molecular spectroscopic database. J. Quant. Spectrosc. Radiat. Transf..

[52] Tu W., Mayne R. (2002). Studies of multi-start clustering for global optimization. Int. J. Numer. Methods Eng..

[53] Ugray Z., Lasdon L., Plummer J., Glover F., Kelly J., Martí R. (2007). Scatter search and local NLP solvers: A multistart framework for global optimization. Inf. J. Comput..

[54] Rinnooy Kan A., Timmer G. (1987). Stochastic global optimization methods part I: Clustering methods. Math. Program..

[55] Dixon L.C.W. (1978). The global optimization problem: An introduction. Towar. Glob. Optim..

[56] Elwakeil O.A., Arora J.S. (1996). Two algorithms for global optimization of general NLP problems. Int. J. Numer. Methods Eng..

[57] Sepulveda A., Epstein L.J. (1996). The repulsion algorithm, a new multistart method for global optimization. Struct. Optim..

[58] Splitter D., Colomer V.B., Neupane S., Chuahy F.D.F., Partridge W.P. In Situ Laser Induced Florescence Measurements of Fuel Dilution from Low Load to Stochastic Pre Ignition Prone Conditions.

[59] Partridge W.P., Choi J.-S., Connatser P.R.M., Currier N., Geckler S., Yezerets A., Kamasamudram K. Cummins/ORNL-FEERC CRADA: NOx Control & Measurement Technology for Heavy-Duty Diesel Engines. 2011 DOE Vehicle Technologies Program Annual Merit Review, Arlington, Virginia, May 12, 2011. https://www.energy.gov/sites/default/files/2014/03/f11/ace032_partridge_2011_o.pdf.

[60] Yoo J., Prikhodko V., Parks J.E., Perfetto A., Geckler S., Partridge W.P. (2015). Fast spatially resolved exhaust gas recirculation (EGR) distribution measurements in an internal combustion engine using absorption spectroscopy. Appl. Spectrosc..

