# Supplementary material for: Development of A Multi-Spectral Pyrometry Sensor for High-Speed Transient Surface-Temperature Measurements in Combustion-Relevant Harsh Environments
Source: Sensors (Basel). 2022 Dec 22;23(1):105. doi: 10.3390/s23010105 (PMC9824859; doi:10.3390/s23010105)
Supplement: Supplementary file 1 [file sensors-23-00105-s001.zip › sensors-2029841-supplementary.pdf]

Supplementary Information File

Development of a multi-spectral pyrometry sensor for high-speed transient surface-temperature measurements in harsh environments

Sneha Neupane[1], Gurneesh Singh Jatana[1,2], Timothy P. Lutz[2], William P. Partridge[*,1]

[1]Oak Ridge National Laboratory, USA
[2]Cummins Inc., USA

[*]Corresponding Author Email: partridgewp@ornl.gov

Table S1: Fitted emissivity coefficients using exponential and linear models

Temperature, °C	Exponential Model			Linear Model		
	a_0	a_1	R^2	a_0	a_1	R^2
598	-2.39	0.43	0.94	0.05	0.08	0.94
649	-2.01	0.44	0.97	0.07	0.12	0.98
702	-1.80	0.43	0.97	0.09	0.15	0.98
751	-1.61	0.38	0.97	0.14	0.14	0.98
801	-1.64	0.44	0.97	0.10	0.18	0.98
852	-1.50	0.42	0.97	0.14	0.19	0.98
907	-1.52	0.44	1.00	0.12	0.20	1.00

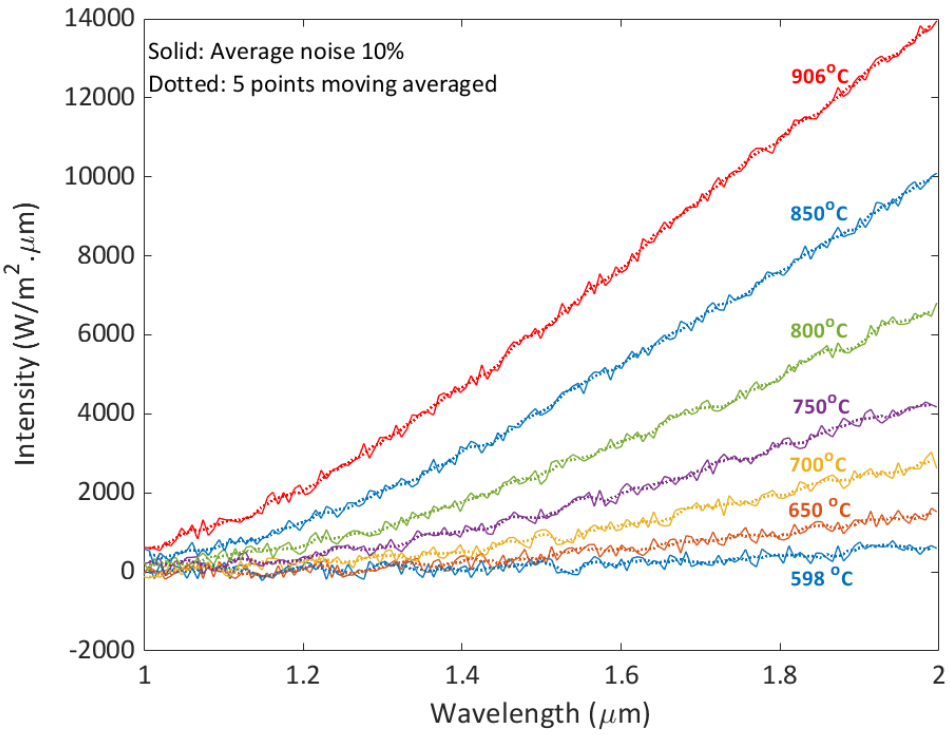

Figure S1. Simulated emission data at seven standard temperatures using Plank's Law, a 1st-order (m=1) polynomial emissivity model, with 10% random noise, and a 5-point moving average.

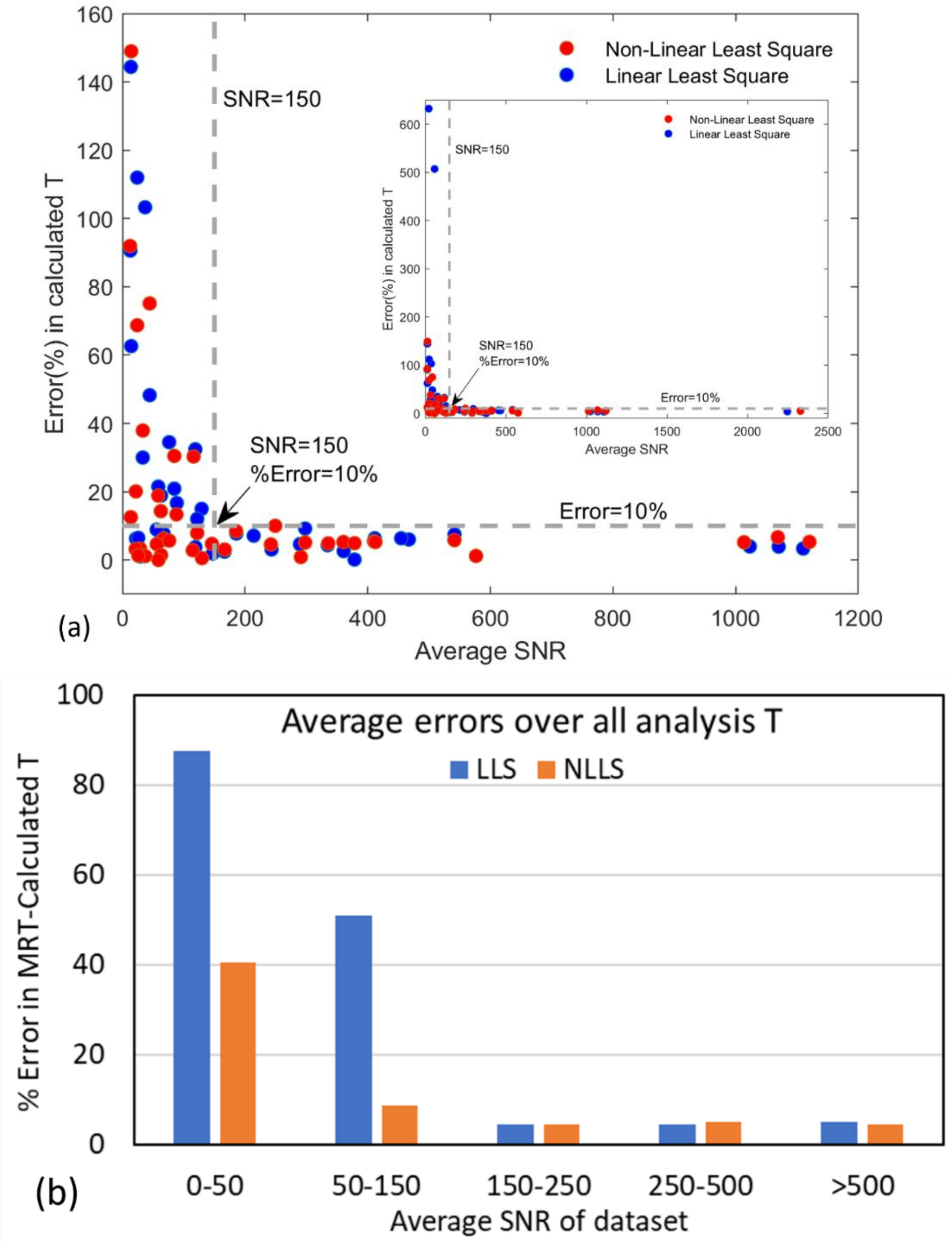

Figure S2. (a) Scatterplot of LLS and NLLS MRT analysis error at 41 SNR levels corresponding to the analysis in Section 3.1. The horizontal dashed line is at 10% temperature error, and vertical dashed lines are shown at SNR of 50 and 150. Inset shows all points, and how both NLLS and LLS MRT have <10% error at very high (~2300) SNR, and how LLS has some very high (~500-630%) at low (~60) SNR. In general, both emissivity models result in <10% temperature error for SNR>150. (b) Average error in MRT-calculated temperature from LLS and NLLS MRT analysis for different SNR regions (W/m^2-μm) using the synthetic surface-emission data.

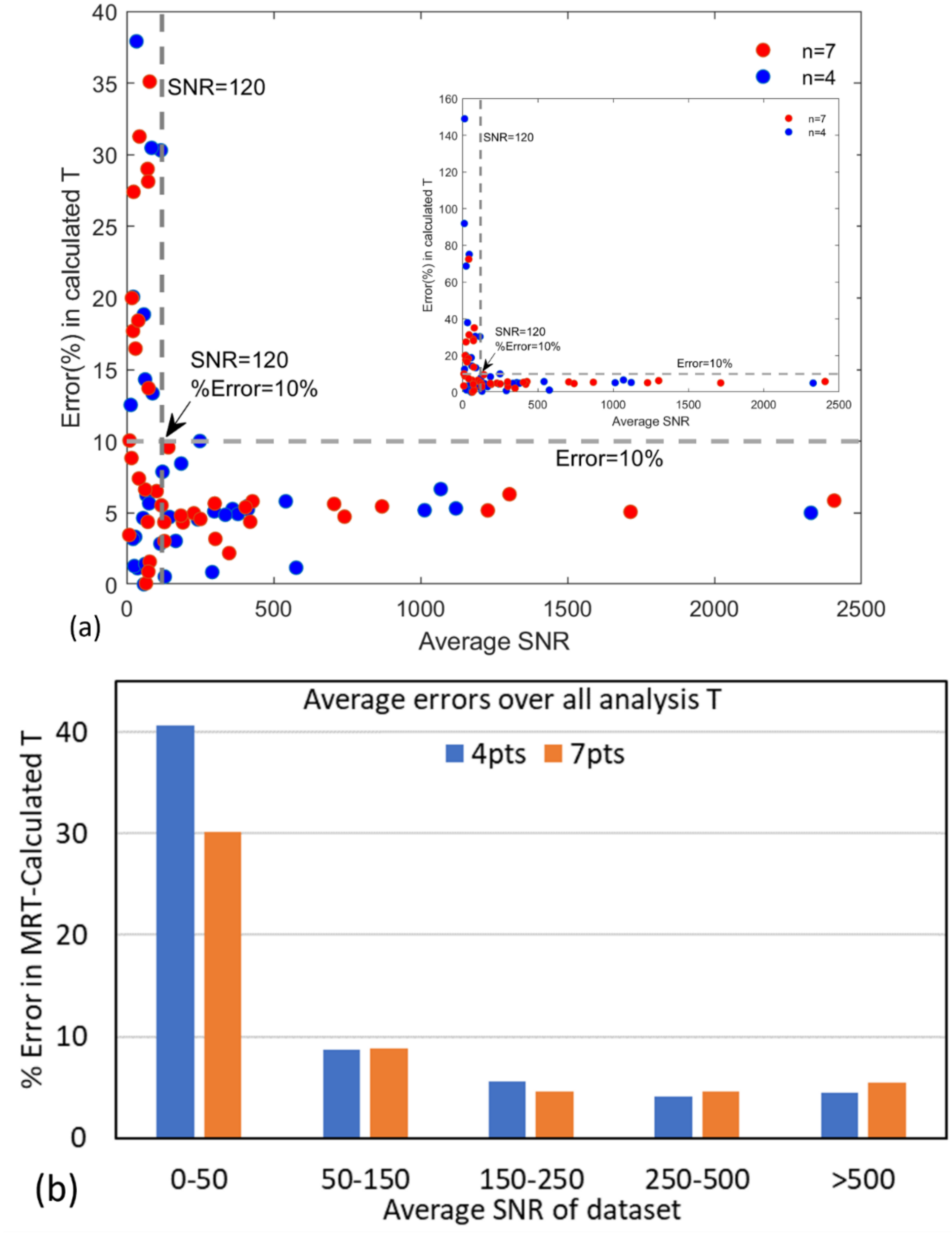

Figure S3. (a) Scatterplot of NLLS MRT analysis error using 4 & 7 spectral channels at 41 SNR levels corresponding to the analysis in Section 4.1. The horizontal dashed line is at 10% temperature error, and vertical dashed lines are shown at SNR of 120. Inset shows all points, and some high (~70-150%) at low (~60) SNR. In general, <10% temperature error is achieved for both 4 & 7 spectral channels for SNR>120. (b) Error in NLLS MRT-calculated temperature using the synthetic data set described in Section 3.1 and four and seven spectral channels (i.e., n=4 & 7) over a range of SNR values (W/m^2-µm).

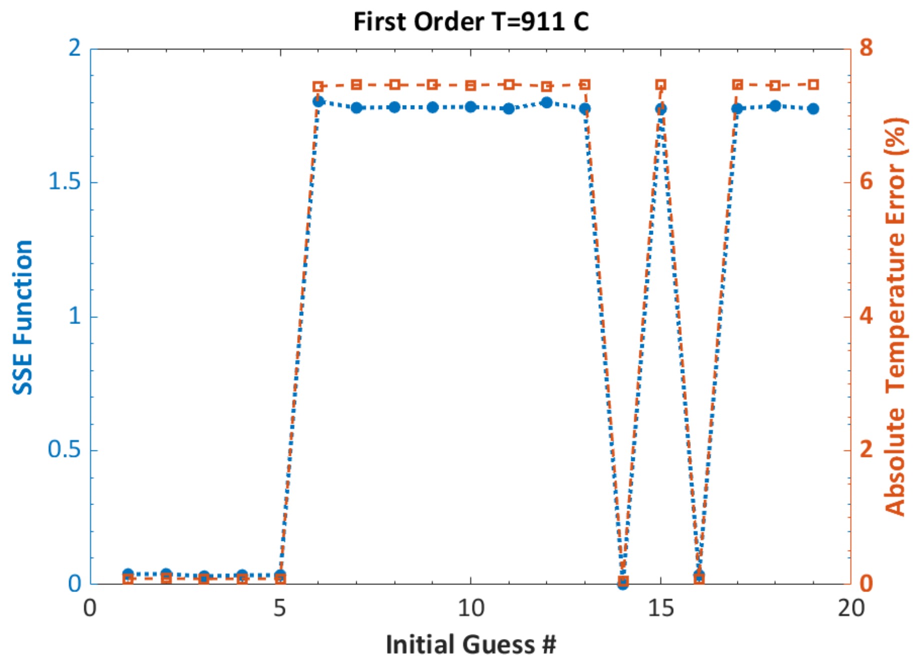

Figure S4. SSE values and corresponding error in calculated temperature using 19 different initial guesses for T. X-axis represents the count for initial guess value of temperature (1 to 19 for T range of 500-1400°C at 50°C interval)

Table S2. Center wavelength (λ_c), full-width at half maximum (FWHM) and source information for the instrument bandpass filters, and focal length (f) and source for the focusing lenses of the instrument.

**Bandpass Filter**			**Focusing Lens**	
λ_c (nm)	FWHM (nm)	**Source**	**f (mm)**	**Source**
1250	50	Edmund, 85-897	25	ThorLabs, LA1252-C
1575	75	SalvoTech, 102386595	25	ThorLabs, LA1252-C
2100	85	SalvoTech, 2020OFS-2100	25	ThorLabs, LA1951-D
3600	140	Andover, 3.60GA05-25	40	ThorLabs, LA5370-E

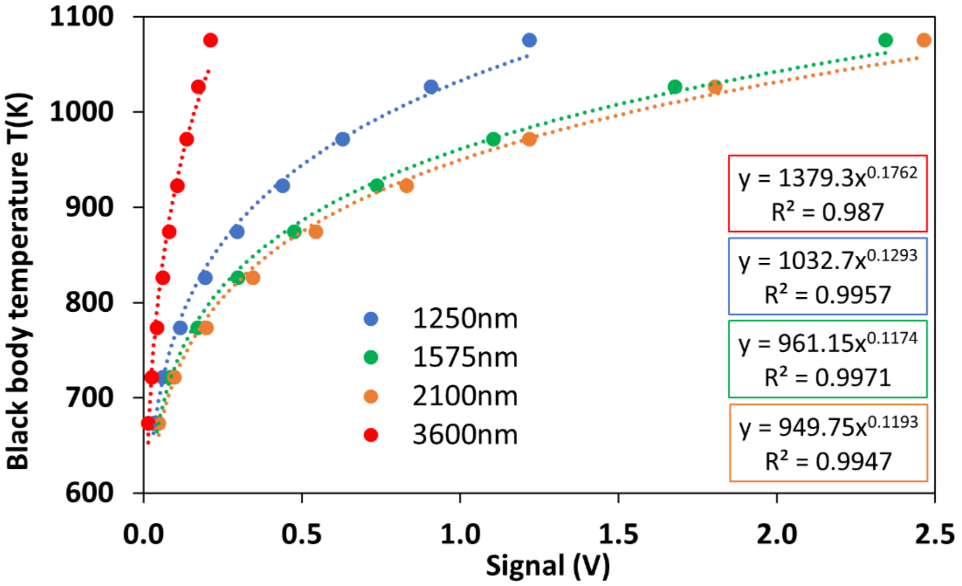

Figure S5. Calibration curves showing signal (V) from each spectral channel at a given black-body temperature (K).

Table S3. Power-law calibration fits for converting measured signal (e.g., S_{1250nm}) at each spectral channel to apparent blackbody temperature (e.g., $T_{a,1250nm}$).

Spectral Channel	Calibration Fit	R^2
1250 nm	$T_{a,1250nm} = 1032.7\ (S_{1250nm})^{0.1293}$	0.9956
1575 nm	$T_{a,1575nm} = 961.15\ (S_{1575nm})^{0.1174}$	0.9970
2100 nm	$T_{a,2100nm} = 949.75\ (S_{2100nm})^{0.1193}$	0.9945
3600 nm	$T_{a,3600nm} = 1379.3\ (S_{3600nm})^{0.1762}$	0.9862

Table S4. Channel-specific and average SNR (W/m^2-µm) values are show. Based on analysis of 20-kHz averaged data, which provides 100-µs resolution or 1.2 cad at 2000 RPM.

True-T (°C)	400	448	501	553	601	698	802
SNR(Average)	63	137	265	488	815	1858	2492
SNR(1250 nm)	53	127	200	371	586	1250	1833
SNR(1575 nm)	117	222	452	805	1459	3509	3343
SNR(2100 nm)	58	117	234	482	748	1820	3300
SNR(3600 nm)	25	84	177	292	467	855	1493
